# Coinfection With SARS-CoV-2 and Influenza A(H1N1) in a Patient Seen at an Influenza-like Illness Surveillance Site in Egypt: Case Report

**DOI:** 10.2196/27433

**Published:** 2021-04-28

**Authors:** Manal Fahim, Hanaa Abu El Sood Ghonim, Wael H Roshdy, Amel Naguib, Nancy Elguindy, Mohamad AbdelFatah, Mohamed Hassany, Amira Mohsen, Salma Afifi, Alaa Eid

**Affiliations:** 1 Department of Surveillance and Epidemiology Ministry of Health and Population Cairo Egypt; 2 Central Public Health Laboratory Cairo Egypt; 3 Ministry of Health and Population Cairo Egypt; 4 Public Health Initiative Cairo Egypt; 5 World Health Organization Egypt Country Office Cairo Egypt; 6 Preventive Sector Cairo Egypt

**Keywords:** influenza-like Illness, pandemic, SARS-CoV-2, COVID-19, influenza, virus, case study, Egypt, flu, coinfection, infectious disease, surveillance, outcome, demographic

## Abstract

**Background:**

Sentinel surveillance of influenza-like illness (ILI) in Egypt started in 2000 at 8 sentinel sites geographically distributed all over the country. In response to the COVID-19 pandemic, SARS-CoV-2 was added to the panel of viral testing by polymerase chain reaction for the first 2 patients with ILI seen at one of the sentinel sites. We report the first SARS-CoV-2 and influenza A(H1N1) virus co-infection with mild symptoms detected through routine ILI surveillance in Egypt.

**Objective:**

This report aims to describe how the case was identified and the demographic and clinical characteristics and outcomes of the patient.

**Methods:**

The case was identified by Central Public Health Laboratory staff, who contacted the ILI sentinel surveillance officer at the Ministry of Health. The case patient was contacted through a telephone call. Detailed information about the patient’s clinical picture, course of disease, and outcome was obtained. The contacts of the patient were investigated for acute respiratory symptoms, disease confirmation, and outcomes.

**Results:**

Among 510 specimens collected from patients with ILI symptoms from October 2019 to August 2020, 61 (12.0%) were COVID-19–positive and 29 (5.7%) tested positive for influenza, including 15 (51.7%) A(H1N1), 11 (38.0%) A(H3N2), and 3 (10.3%) influenza B specimens. A 21-year-old woman was confirmed to have SARS-CoV-2 and influenza A(H1N1) virus coinfection. She had a high fever of 40.2 °C and mild respiratory symptoms that resolved within 2 days with symptomatic treatment. All five of her family contacts had mild respiratory symptoms 2-3 days after exposure to the confirmed case, and their symptoms resolved without treatment or investigation.

**Conclusions:**

This case highlights the possible occurrence of SARS-CoV-2/influenza A(H1N1) coinfection in younger and healthy people, who may resolve the infection rapidly. We emphasize the usefulness of the surveillance system for detection of viral causative agents of ILI and recommend broadening of the testing panel, especially if it can guide case management.

## Introduction

### COVID-19 Epidemic Situation

COVID-19 is caused by the virus SARS-CoV-2. As of September 7, 2020, a total of 27,314,629 confirmed COVID-19 cases and 893,474 related deaths had been reported worldwide [1]. In Egypt, a total of 99,863 confirmed COVID-19 cases and 5530 related deaths had been reported as of September 7, 2020 [2].

### Egypt Influenza-like Illness Surveillance

Influenza-like illness (ILI) sentinel surveillance in Egypt started in 2000 at 8 sentinel sites geographically distributed all over the country. Patients presenting to the outpatient clinics in the participating hospitals with fever and cough within the last 10 days are required to provide throat swabs to be maintained in viral transport media, stored in a nitrogen tank at –80 ⁰C and shipped on a weekly basis to the Central Public Health Laboratory (CPHL) in Cairo for testing for influenza type and subtype by reverse transcriptase–polymerase chain reaction (RT-PCR). Demographic and clinical data of the patients are collected in a special database that is regularly analyzed. Reports of the rate of influenza positivity and prevalent influenza types and subtypes are provided to decision makers and relevant stakeholders on a weekly basis.

### Modifications to the ILI Surveillance Scheme During the COVID-19 Pandemic

Since the beginning of the COVID-19 pandemic, the Egyptian Ministry of Health and Population (MoHP) requested that all patients with acute respiratory symptoms at all governmental hospitals be assessed by the emergency department (ED). Accordingly, ILI surveillance teams were requested to enroll the first 2 patients with ILI symptoms every day in the ED and follow the usual surveillance methodology for data and sample collection. The MoHP requested that SARS-CoV-2 be added to the testing panel at all ILI surveillance sites.

Early in the pandemic, CPHL was the only laboratory approved by the MoHP for SARS-CoV-2 testing. Because of resource constraints, testing for influenza was placed on hold starting in October 2019, and specimens collected from ILI patients were archived at –70 °C for subsequent testing when possible. As the number of COVID-19 patients in Egypt started to decline in August 2020, CPHL began to test the archived specimens collected at ILI sites.

### Study Objectives

On August 16, 2020, CPHL notified the MoHP surveillance department of a case with mixed SARS-CoV-2 and influenza A(H1N1) virus infection. This report aims to describe how the case was identified and to describe the patient’s demographic and clinical characteristics and outcomes.

## Methods

### ILI Surveillance Methods: Case Detection

The influenza virological surveillance was implemented in Egypt in 2000 at 8 outpatient clinics in 6 governorates across Egypt. Participants enrolled in the virological surveillance (2-3 ILI subjects per day, 6 days per week) are interviewed to obtain their demographic information. The World Health Organization surveillance standards for ILI are used to recruit patients, including abrupt onset of fever ≥38 ºC with respiratory manifestations of cough with onset within the last 10 days [3].

While testing routine ILI samples, CPHL staff noted a case of coinfection of SARS-CoV-2 and influenza A(H1N1), confirmed by PCR testing.

### Case Investigation

The CPHL staff contacted a surveillance officer, who investigated the case through a telephone call. The clinical picture, disease course, severity risk factors, other clinical investigations and disease outcome were investigated for the case patient and her contacts. Surveillance data were entered in a real-time web-based database at MoHP, and laboratory data were entered at CPHL to be merged automatically with the surveillance data. ILI data from October 2019 to August 2020 were extracted and analyzed for influenza and SARS-CoV-2 as well as their coinfection.

### Laboratory and Clinical Investigations

A nasopharyngeal swab was collected from the patient, and nucleic acid extraction for the clinical sample was performed using the chemagic 360 instrument (PerkinElmer Inc). SARS-CoV-2 RNA (ORF1ab) was detected using a VIASURE SARS-CoV-2 Real-Time PCR Detection Kit (Certest Biotec SL). The RT-PCR runs were performed in triplicate and according to the manufacturer’s recommendations, and the samples were confirmed to be positive for SARS-CoV-2 using a cobas 6800 system (Roche Holding AG). Moreover, influenza A(H1N1) was tested by real-time PCR using the US Centers for Disease Control protocol [4]. A complete blood count (CBC) and computed tomography (CT) images of the chest were obtained for the patient.

## Results

### ILI Surveillance Virological Results

**Figure 1:**
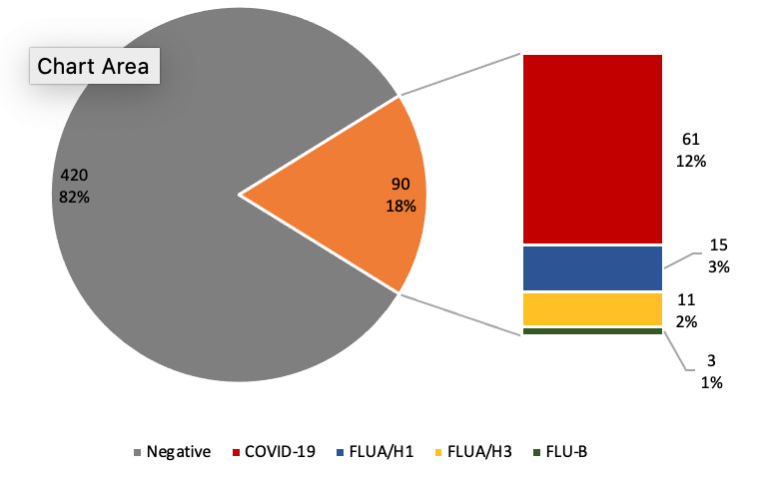
Viral causes of 510 specimens collected from influenza-like illness sentinel surveillance in Egypt from October 2019 to September 2020. FLUA/H1: influenza A(H1N1); FLUA/H3: influenza A(H3N2); FLU-B: influenza B.

Among 510 specimens collected from patients with ILI symptoms from February to August 2020, 29 (5.7%) were positive for influenza. Of those, 15 (51.7%) were positive for A(H1N1), 11 (38.0%) for A(H3N2), and 3 (10.3%) for influenza B. The first case of COVID-19 in Egypt was announced on February 14, whereas the ILI surveillance identified its first COVID-19 case 2 weeks later, announcing the beginning of community transmission of the disease in Egypt. Of the 510 specimens tested, 61(12.0%) were COVID-19–positive (Figure 1). One case was confirmed to have both SARS-CoV-2 and influenza A(H1N1).

### Case Investigation Results

The case patient presented to the outpatient clinic of one of the ILI surveillance sites that serves Helwan, a semiurban area in Cairo, on May 2020. She was a 21-year-old female student complaining of fever, cough, fatigue, and malaise for 2 days with no other symptoms or associated comorbidities. She presented with a high fever of 40.2°C, and her chest was free on auscultation. The patient’s CBC was normal and her chest CT imaging was clear, indicating that she had no lower respiratory tract infection. The patient was swabbed and sent home for treatment; she was given symptomatic treatment in the form of an antipyretic, an antitussive, and oral cefadroxil 2 g per day. Her symptoms persisted for 2 days, followed by full recovery. At home, no isolation was performed for the case patient, and 4 of her 5 family contacts had mild respiratory symptoms 2-3 days after exposure to the confirmed case. Secondary cases included the 2 parents (both 49 years of age) and 2 brothers (9 and 16 years of age); all of them recovered within 2-3 days except for the case patient’s father, who experienced hypertension and recovered in 2 weeks. None of the case patient’s contacts sought health care, and they all recovered without treatment.

### Laboratory and Clinical Investigation Results

The patient tested positive by RT-PCR for both SARS-CoV-2 and influenza A(H1N1). The main cycle threshold (Ct) value for the SARS-CoV-2 N gene was 16.1, and that for ORF1ab was 14.2 (Certest Biotec SL); also, the main Ct value for the SARS-CoV-2 ORF1ab gene was 14.9 and that for the E gene was 15.6 (cobas 6800, Roche Holding AG) as a confirmatory method. At the same time, the main Ct value for influenza A was 32.6, that for swFluA was 32.2, and that for swH1 was 31.6. The case patient’s CBC was normal, and her chest CT imaging was clear.

## Discussion

### Coinfection of SARS-CoV-2 and Influenza A(H1N1)

This study illustrates the characteristics of the first case of SARS-CoV-2 and influenza A(H1N1) coinfection with mild ILI symptoms in Egypt and highlights the benefit of a surveillance system for codetection of respiratory viruses.

Coinfection of other coronaviruses and influenza A viruses has been reported [5]. During the current COVID-19 pandemic, coinfection of SARS-CoV-2 and influenza A(H1N1) was reported in case studies conducted in many countries, including China, Italy, Iran, and Japan [6-9].

### Predominant Viral Cause

The abrupt symptom of high fever, short secondary incubation period, and mildness and short course of disease suggest that influenza was the main causative agent [10]. Interestingly, the Ct values in the specimen of this patient indicate that the viral load of SARS-CoV-2 infection was much higher than that of influenza A(H1N1). Studies suggest that the viral load of SARS-CoV-2 peaks around symptom onset or a few days later [11]. This suggests that influenza infection occurred earlier and competitively suppressed replication of SARS-CoV-2 [12].

### How the Egyptian Case Compares to Cases Reported From Other Countries

#### Demographics

The case patient reported in Egypt was a young woman, whereas most of the patients reported from other countries were older. In a mini-review by D’Abramo et al [7] that describes 37 patients with SARS-CoV-2 and influenza coinfection, it was found that 66.7% of patients were ≥50 years of age, and 56.5% were male.

#### Disease Course and Severity

Most of the cases of coinfection reported from other countries had a prolonged course of the disease, and all of them were admitted to hospital [6-10]. Although the case patient reported from Egypt had mild symptoms, she was detected during routine ILI surveillance activities. She had a short disease course of 4 days with home treatment, and her CBC and CT chest imaging were normal. Her contacts had even milder symptoms; therefore, they did not seek any medical advice.

#### Predisposing Factors

Most of the reported cases with coinfection had predisposing factors reducing their immunity, and many of them required mechanical ventilation or intensive care unit (ICU) admission [6-9,13]. It was found that more than 60% of patients with coinfection had comorbidities, 33% needed artificial ventilation, and 29% were admitted to the ICU. To date, the case reported from Egypt is the only one from any country with mild upper respiratory symptoms. This could be related to the patient’s age and gender in addition to the absence of predisposing comorbidities.

The results of ILI patient testing indicated that more than 80% of cases were negative for both SARS-CoV-2 and influenza. Broader viral testing may be needed to identify the etiology, particularly if it would affect patient treatment [13].

### Conclusion

Egypt is reporting a case of SARS-CoV-2 and influenza A(H1N1) co-infection with mild ILI symptoms. This finding suggested that coinfection can occur in people of younger age with no comorbidities. The report showed that patient immunity can overcome both infections, leading to full recovery in a short period with no need for medical procedures. ILI surveillance proved effective in the detection of the viral causes of patients with ILI symptoms. Broadening of the testing panel is recommended, especially if it could guide improvement of case management guidelines.

## Abbreviations

CBC: complete blood count
CPHL: Central Public Health Laboratory
Ct: cycle threshold
CT: computed tomography
ICU: intensive care unit
ILI: influenza-like illness
MoHP: Ministry of Health and Population
RT-PCR: reverse transcriptase–polymerase chain reaction
